# Otariid gammaherpesvirus 1 in South American fur seals (*Arctocephalus australis*) and a novel related herpesvirus in free-ranging South American sea lions (*Otaria byronia*): Prevalence and effects of age, sex, and sample type

**DOI:** 10.1371/journal.pone.0299404

**Published:** 2024-03-06

**Authors:** Karisa N. Tang, Michael J. Adkesson, Susana Cárdenas-Alayza, Laura Adamovicz, Alissa C. Deming, James F. X. Wellehan, April Childress, Galaxia Cortes-Hinojosa, Kathleen Colegrove, Jennifer N. Langan, Matthew C. Allender

**Affiliations:** 1 Chicago Zoological Society, Brookfield Zoo, Brookfield, IL, United States of America; 2 Illinois Zoological and Aquatic Animal Residency, Urbana, IL, United States of America; 3 A. Watson Armour III Center for Animal Health and Welfare, John G. Shedd Aquarium, Chicago, IL, United States of America; 4 Centro para la Sostenibilidad Ambiental, Universidad Peruana Cayetano Heredia, Lima, Peru; 5 Departamento de Ciencias Biológicas y Fisiológicas, Facultad de Ciencias y Filosofía, Universidad Peruana Cayetano Heredia, Lima, Peru; 6 Wildlife Epidemiology Laboratory, University of Illinois College of Veterinary Medicine, Urbana, IL, United States of America; 7 Pacific Marine Mammal Center, Laguna Beach, CA, United States of America; 8 Department of Comparative, Diagnostic, and Population Medicine, College of Veterinary Medicine, University of Florida, Gainesville, FL, United States of America; 9 Escuela de Medicina Veterinaria, Facultad de Agronomía e Ingeniería Forestal, Facultad de Ciencias Biológicas y Facultad de Medicina, Pontificia Universidad Católica de Chile, Santiago, Chile; 10 Zoological Pathology Program, Veterinary Diagnostic Laboratory, College of Veterinary Medicine, University of Illinois, Brookfield, IL, United States of America; 11 Department of Veterinary Clinical Medicine, College of Veterinary Medicine, University of Illinois, Urbana, IL, United States of America; Animal Health Centre, CANADA

## Abstract

Otariid gammaherpesvirus 1 (OtGHV1) is associated with high rates of urogenital carcinoma in free-ranging California sea lions (*Zalophus californianus*; CSL), and until recently was reported only in the Northern Hemisphere. The objective of this study was to survey free-ranging South American sea lions (*Otaria byronia; SASL*) and South American fur seals (*Arctocephalus australis*: *SAFS*) in Punta San Juan, Peru for OtGHV1 and to determine prevalence characteristics. Twenty-one percent (14/67) of urogenital swabs collected over three years (2011, 2014, 2015) from live pinnipeds of both species tested positive with a pan-herpesvirus conventional PCR. Sequencing of SAFS amplicons revealed 100% homology to OtGHV1 at the DNA polymerase, glycoprotein B, and viral *bcl2*-like genes. Sequencing of SASL amplicons revealed a novel related virus, herein called Otariid gammaherpesvirus 8 (OtGHV8). For comparison of sample sites, urogenital, conjunctival, and oropharyngeal swabs collected from 136 live pinnipeds of both species at Punta San Juan between 2011–2018 were then assayed using quantitative PCR for a segment of the OtGHV1/8 DNA polymerase gene using a qPCR assay now determined to cross-react between the two viruses. In total, across both species, 38.6% (51/132) of urogenital swabs, 5.6% (4/71) of conjunctival swabs, and 1.1% (1/90) of oropharyngeal swabs were positive for OtGHV1/8, with SASL only positive on urogenital swabs. Results from SASL were complicated by the finding of OtGHV8, necessitating further study to determine prevalence of OtGHV1 versus OtGHV8 using an alternate assay. Results from SAFS suggest a potential relationship between OtGHV1 in SAFS and CSL. Though necropsy surveillance in SAFS is very limited, geographic patterns of OtGHV1-associated urogenital carcinoma in CSL and the tendency of herpesviruses to cause more detrimental disease in aberrant hosts suggests that it is possible that SAFS may be the definitive host of OtGHV1, which gives further insight into the diversity and phyogeography of this clade of related gammaherpesviruses.

## Introduction

Worldwide, many ecosystems have undergone unprecedented change over the past 150 years due to anthropogenic activity. South American pinniped populations have experienced declines related to hunting, habitat encroachment, overfishing, and pollution [1–3]. Coastal development reduces available habitat and subsequently increases intra- and inter-specific contact between animals, a setting that creates the potential for increased disease transmission [4–6].

The Punta San Juan marine protected area (PSJ) guards critical rookeries for two pinniped species considered endangered by the Peruvian government: the South American sea lion (*Otaria byronia*, SASL) and the South American fur seal (*Arctocephalus australis*, SAFS) [1, 2]. Commercial hunting in the early 1900s decimated SAFS and SASL populations, and subsequent depletion of fish stocks by commercial fisheries and alterations in prey availability from El Niño Southern Oscillation (ENSO) events have limited population recovery [7, 8]. Surveillance for some infectious diseases has been conducted in female SAFS [9, 10], and additional studies are ongoing in both SAFS and SASL to assess the role of disease in population health.

The family Otariidae separated from their closest relatives, the Odobenidae, approximately 19.4 million years ago (mya) [11]. Within Otariidae, the earliest extant lineage to diverge (approximately 9 mya) contains the northern fur seal (*Callorhinus ursinus*, NFS) [11]. There was then a divergence between the species now in the southern hemisphere (*Arctocephalus*, *Otaria*, *Phocarctos*, *Neophoca*) and the remaining northern species (*Zalophus*, *Eumetopias*) approximately 5.4 mya [11, 12]. The genera and species of the southern hemisphere diverged in a relatively brief time period, from 3 mya to 2.5 mya. It has been hypothesized that global cooling and associated increased oceanic productivity at this time enabled dispersal across the equator that had not been possible in the earlier warmer period 5 mya-3.5 mya, and rapid diversification occurred as the southern clade accessed new habitats [11]. Within SAFS, the Peruvian population has distinct morphological, molecular and biological features making them genetically distinct and have been proposed as an Evolutionary Significant Unit, subspecies and recently a new species that diverged ~0.64 mya, although official recognition and an accompanying nomenclature change has not yet occurred [2, 13–16]. These phylogenetic relationships and geographic dispersal of species is relevant when considering disease transmission and differing clinical responses to disease between species.

Herpesviruses, a family of large DNA viruses with intranuclear replication, usually display high host fidelity and appear to have often codiverged with the evolution of their host species [17, 18]. Herpesviruses have adapted to their endemic hosts over millions of years. Disease is typically relatively mild in the absence of immunosuppression, with latency being a characteristic of endemic herpesviral infection. When endemic hosts are immunosuppressed, herpesviruses can be oncogenic [19]. In aberrant hosts, the complex balance needed for latency is not present, and rapidly fatal fulminant disease is often seen [20, 21]. This more rapid fulminant disease may be neoplastic [22, 23]. Host jumps are generally within the same host superorder, and more rapid, severe disease may be a significant barrier to establishment of a herpesvirus in a novel host species.

There are currently eight described otariid herpesviruses, seven in the subfamily *Gammaherpesvirinae*, and one in the subfamily *Alphaherpesvirinae*. Otariid Alphaherpesvirus 1 was described in a SASL with no associated viral lesions [24]. Otariid gammaherpesvirus 1 (OtGHV1) is well documented in California sea lions (*Zalophus californianus*, CSL) and has been closely associated with development of urogenital carcinoma [25, 26]. OtGHV2, 3, 4, 5, 6, and 7 have also been identified in otariid species, but their impact on health is not well understood [27–33]. These seven gammaherpesviruses form three clades, one containing OtGHV1 and 4 from CSL and NFS, respectively, one containing OtGHV3 from CSL in a clade utilizing hosts in the Caniformia including several phocids [34], and one in the genus *Percavirus* containing OtGHV2, 5, 6, and 7 [35]. While OtGHV2 is described in CSL, OtHV5 and 6 were initially sequenced from SAFS in Brazil. These viruses have also been detected in subantarctic fur seals (*Arctocephalus tropicalis*, SubFS), the host where OtGHV7 was also found [33, 35]. SubFS breed on islands north of the Antarctic convergence in the eastern hemisphere, but occasional vagrants are found in South America, where they may interact with SAFS [36]. Finding two gammaherpesviruses (OtHV5 and 6) each in two distinct otariid host species (SAFS & SubFS) is notable. Of the sequences available in GenBank, 2 of 3 OtGHV5 sequences are from SubFS, and 3 of 4 OtGHV6 sequences are from SAFS; potentially indicating that these viruses are endemic in the species where they are most commonly found. The only lesions that were reported as consistent with viral etiology associated with either OtGHV5 or 6 were skin ulcerations in SAFS which contained OtGHV5 [32]; viral associated lesions in SAFS would be more expected if OtGHV5 were a more recent introduction from SubFS.

OtGHV1 was found in over 20% of subadult and adult necropsied CSL in California from 1979–1994 and 2005–2015 [37, 38]. There are several candidate oncogenes in OtGHV1 that are expressed at high levels in tumors, including a viral *bcl2*-like gene [39]. The virus is suggested to be transmitted sexually based on a higher prevalence in urogenital tissues of adult males [40, 41]. OtGHV1-associated metastatic urogenital carcinoma is largely restricted to CSL, but has been documented in a single SAFS housed in captivity in Europe that was not housed with CSL [42]. Most cancers result from a multistep process involving several contributing factors in the progression of cellular transformation to malignancy. Co-factors suggested to play a role in carcinogenesis in CSL [25, 43] include contaminants [44], host genetic variability [45], endogenous hormone disruption [46], and co-infections with bacteria [47]. Exposure to organic environmental contaminants (polychlorinated biphenyls, organochlorine pesticides, and polybrominated diphenyl ethers) at PSJ was documented to be at low levels not associated with detrimental health effects in Humboldt penguins (*Spheniscus humboldti*), suggesting that environmental toxins are not a present concern in the sympatric pinniped species [48].

To further illustrate the complexity in development of carcinoma, OtGHV1-positive CSL have been identified with PCR in the Gulf of California population without published reports of urogenital carcinoma, although necropsy surveillance of stranded animals is lacking [41]. Northern fur seals (*Callorhinus ursinus*, NFS) have an overlapping geographic range with CSL in California, but OtGHV1 and OtGHV1-associated urogenital carcinoma has not been identified despite large-scale surveillance; it is hypothesized that OtGHV4, endemic in NFS and closely related to OtGHV1, may provide immunologic cross-protection [27]. Additional surveillance for OtGHV1 in other species and geographic locations may further elucidate the role this virus plays in development of neoplasia. Given the scarce information in pinniped populations on the south Pacific, sampling sympatric pinnipeds in PSJ is an excellent opportunity to detect and characterize the epidemiology of herpesviruses in Peru. This may be important in starting to further assess presence of ecologically-relevant clinical disease, as well as potential relationships between herpesviruses seen in related pinnipeds across North and South America. The objectives of this study were to (1) survey for OtGHV1 in SASL and SAFS at PSJ, (2) describe the prevalence of OtGHV1 in the populations, and (3) evaluate associations between OtGHV1 detection and species, sex, age class, and sampling site.

## Materials and methods

### Sample collection

SAFS and SASL had been captured in 2011 and 2014–2018 for health evaluation at PSJ, Peru (15°22’S, 75°12’W) under Peruvian permits RJ 23–2011, 024–2014, 229–2015, and 019-2016-SERNANP-RNSIIPG. All sample collection was conducted in November, except for 2017 (February) and 2018 (April). Adult animals were anesthetized under the supervision of a veterinarian. Pups under 6 months of age were either manually restrained or anesthetized based on protocol needs for concurrent projects. A sterile cotton-tipped applicator (Cardinal Health sterile cotton tipped applicator with plastic shaft, Cardinal Health, Dublin, Ohio) was used to collect urogenital swabs (URO) from the prepuce or vulva, conjunctival swabs (CON) from the inner palpebrae, and oropharyngeal swabs (ORO) from the dorsal aspect of the oral cavity along the soft palate. All swabs were placed without media in microcentrifuge (Eppendorf PCR tubes, Eppendorf North America, Hauppauge, NY) tubes and promptly placed on ice. Samples were frozen within 12 hours and were maintained at -80°C until DNA extraction.

### Conventional PCR

DNA was extracted according to the manufacturer’s instructions using a commercially available extraction kit (QIAamp DNA Blood Mini Kit, Qiagen, Valencia, CA). DNA quantity and purity were assessed using a spectrophotometer (Nanodrop spectrophotometer, Thermo Scientific, Wilmington, DE). Conventional PCR was performed using a nested pan-herpesvirus consensus assay targeting a short (~200bp) segment of the DNA polymerase gene [49]. Positive samples were treated with ExoSAP-IT (USB Corporation, Cleveland, OH) or purified from isolated gel slices (QIAquick Gel Extraction Kit, Qiagen, Valencia, CA) and commercially sequenced in both directions (ACGT Inc, Wheeling, IL). Sequences were compared to known sequences in GenBank using BLASTN [50].

To obtain additional sequence from thirteen SAFS URO samples and one SASL URO sample, two additional PCR assays were designed using primers OtHV1_4GPF (5’-ATGAAGCACAACTGATAGAC-3’) and OtHV1_4GPR (5’-CATTCCACTCAGTTTCATTA-3’) targeting glycoprotein B, and primers OtHV1_4.bcl2F (5’-CGGATGCTTCAACTAGCACA-3’) and OtHV1_4.bcl2R (5’- CGATGACACCAGTTCTTCCA-3’) targeting the less conserved 5’ portion of a *bcl2*- like gene found in OtGHV1 and OtGHV4. Reactions were amplified in a thermal cycler (Px2, Thermo Fisher Scientific, Inc., Waltham, MA) using Platinum® Taq DNA Polymerase (Invitrogen Corp., Carlsbad, CA) in 30μL reactions following manufacturer’s instructions. Amplification conditions were as follows: initial denaturation at 94°C for 5 min; 45 cycles of amplification with each cycle consisting of denaturation at 94°C for 30 s, annealing at 46°C for 1 min, and elongation at 72°C for 1 min; a final elongation step was performed at 72°C for 7 min followed by a 4°C hold. PCR products were resolved on 1–1.5% agarose gels. Bands were excised and purified using a QIAquick Gel Extraction Kit (Qiagen Inc.). Direct sequencing was performed (BigDye Terminator v3.1 Kit, Applied Biosystems Inc., Foster City, CA).

### Quantitative PCR

DNA extracts from the swabs were assayed using a Taqman quantitative PCR (qPCR) assay for OtGHV-1, as previously described [39]. Real-time qPCR was performed using a real-time PCR thermocycler (7500 ABI real-time PCR System, Applied Biosystems, Carlsbad, CA), and resulting data were analyzed using Sequence Detection Software (Applied Biosystems, Carlsbad, CA). All reactions, including positive and negative controls, were run in triplicate. Positive samples were quantified using standard curve from 10^1^–10^7^ target gene copies per reaction. Samples were considered positive if cycle threshold values for all replicates were less than those from the lowest standard dilution. Resulting OtGHV-1 copy numbers were then standardized based on the concentration of DNA in each sample. Final results are reported as OtGHV-1 copy numbers per ng of DNA.

### Statistical analysis

#### Prevalence data

Prevalence of Otarine herpesvirus in URO, CON, and ORO swabs using qPCR was calculated. Continuous variables (viral copy number) were tested for normality using the Shapiro-Wilk test. Descriptive statistics including median, 10–90% percentiles, and minimum and maximum were tabulated. Multivariable logistic regression models were built separately for each species with herpesvirus qPCR detection status (positive or negative) as the outcome variable and the categorical predictor variables of age class, sex, and year sampled. All models were fitted using the glm function in R studio version 1.0.136 at an alpha value of 0.05 (R Core Team 2018) [51]. An information theoretic approach was used to determine which model from the candidate set performed best using the AICcmodavg package in R studio [52, 53]. Odds ratios (OR) were calculated from the coefficients of the highest-ranking model. The Mann-Whitney U test was used to determine differences in copy number between categorical variables which were significantly associated with qPCR detection status, or those which approached statistical significance (P < 0.1) in the logistic regression model using commercially available software (IBM SPSS Statistics 24.0, IBM, Chicago, IL).

#### Agreement analysis

Agreement between qPCR results from different body tissues (CON, URO, ORO) was assessed using Cohen’s kappa. Interpretation of the kappa statistic was performed using previously established criteria for no agreement (0–0.2), minimal (0.21–0.39), weak (0.4–0.59), moderate (0.6–0.79), strong (0.8–0.9), and almost perfect (>0.9) agreement [54].

#### Phylogenetic analysis

The predicted homologous 229–282 amino acid (AA) sequences of 66 herpesvirus DNA-dependent-DNA polymerases were downloaded from GenBank (type species were selected when possible) and aligned using MAFFT [55]. Partial homologous amino acid sequences for which full-length data was not available were included, with ambiguities added for unknown AA. Predicted homologous 100–104 AA sequences of 41 herpesvirus glycoprotein B were similarly aligned. Chelonid Alphaherpesvirus 5 (GenBank accession # YP_009207091 for polymerase, YP_009207088 for glycoprotein B) was selected as the outgroup. Bayesian analyses of each alignment were conducted using Mr. Bayes 3.2.7 on the CIPRES server with mixed amino acid substitution models, gamma distributed rate variation, and a proportion of invariable sites [56, 57]. A total of 4 chains were run, and statistical convergence was assessed via the average standard deviation of split frequencies and potential scale reduction factors of parameters. The initial 25% of 2,000,000 iterations were discarded as burn in. The *bcl2*-like gene did not have sufficient homology over the region amplified for reliable alignment and phylogenetic analysis.

## Results

### Sample population

A total of 132 animals (81 South American sea lions and 51 South American fur seals) were sampled over six years; the distributions by year, species, sex, age class, and sample type are provided in Table 1. There were 132 URO swabs, 71 CON swabs, and 90 ORO swabs available for analysis, as not every animal had each body site sampled.

**Table 1:** Sample size of urogenital, conjunctival, and oropharyngeal swabs taken from adult South American sea lions (*Otaria byronia*) and South American fur seals (*Arctocephalus australis*) of both sexes over five years in Punta San Juan, Peru.

Year	Species^a^	Sex	Age class	Urogenital swabs	Conjunctival swabs	Oropharyngeal swabs
2011	SAFS	Female	Adult	31	32	32
	SASL	Male	Adult	7	8	7
2014	SAFS	Male	Adult	6	6	6
	SASL	Male	Adult	5	6	6
2015	SAFS	Female	Adult	8	9	9
	SASL	Male	Adult	9	9	9
2016	SAFS	Male	Adult	6	0	6
2017	SASL	Female	Adult	15	0	14
2018	SASL	Female	Adult	8	0	0
	SASL	Female	Pup	11	0	0
	SASL	Male	Pup	25	0	0

^a^SAFS = South American fur seal (*Arctocephalus australis*), SASL = South American sea lion (*Otaria byronia*)

### Prevalence data

#### Conventional PCR

Conventional consensus PCR targeting the DNA polymerase gene was performed on 67 URO swabs (22 adult SASL, 45 adult SAFS) from the years 2011, 2014, and 2015 to screen for herpesviruses. A sample was considered positive if it had a band of equivalent size to the positive control (Table 2).

**Table 2:** Descriptive statistics for prevalence of Otarine herpesvirus 1/Otarine herpesvirus 8 using PCR in South American fur seals (*Arctocephalus australis*; SAFS) and South American sea lion (*Otaria byronia;* SASL) in Peru.

		Sample Type	Number Positive	Prevalence	95% CI Prevalence	Median Copy number (copies/ng DNA)	Min copy	Max copy
Conventional PCR								
	SASL	Urogenital swab	1	5%	NA	NA		
	SAFS	Urogenital swab	13	29%	NA	NA		
Quantitative PCR	SAFS	Urogenital swab	19	37.20%	24.1–51.9%	98,314	18.4	341,325,792
	SAFS	Conjunctival swab	4	8.50%	2.4–20.4%	213	164	2025
	SAFS	Oronasal swab	1	1.90%	0.5–10.1%	274	NA	NA
	SASL Adults/subadults	Urogenital swab	27	61.4%%	45.5–75.6%	22,573	103	35,444,317
	SASL pups/yearlings	Urogenital swab	5	13.50%	4.5–28.8%	157	91	1474

Four SAFS URO samples were sequenced further, amplification of partial DNA polymerase resulted in identical 166 bp sequences after primers were edited out that had 100% nucleotide homology with OtGHV1 (GenBank # MN334559) and 95.18% nucleotide homology with OtGHV4 (GenBank # MN545486). From the one SASL URO sample examined further, amplification of partial DNA polymerase resulted in a 166 bp sequence after primers were edited out that had 94.58% nucleotide homology with OtGHV1 (GenBank # MN334559) and 93.37% nucleotide homology with OtGHV4 (GenBank # MN545486). Further amplification of the SASL URO sample using the outer primers of the nested polymerase protocol resulted in a 472 bp sequence after primers were edited out that had 95.97% nucleotide homology (96.18% predicted amino acid homology) with OtGHV1 (GenBank # MN334559) and 95.13% nucleotide homology (95.33% predicted amino acid homology) with OtGHV4 (GenBank # MN545486).

Partial glycoprotein B was amplified from nine URO samples, resulting in identical 311 bp sequences after primers were edited out that had 99.36% nucleotide homology (100% predicted amino acid homology) with OtGHV1 (GenBank # MN334559) and 95.18% nucleotide homology (94.17% predicted amino acid homology) with OtGHV4 (GenBank # MN545486). From the one SASL URO sample examined further, amplification of partial DNA polymerase resulted in a 311 bp sequence after primers were edited out that had 97.10% nucleotide homology (98.06% predicted amino acid homology) with OtGHV1 (GenBank # MN334559) and 93.49% nucleotide homology (94.17% predicted amino acid homology) with OtGHV4 (GenBank # MN545486).

Partial *bcl2*-like gene was amplified from five URO samples, resulting in identical 223 bp sequences after primers were edited out that had 100% nucleotide homology with OtGHV1 (GenBank # MN334559) and 90.27% nucleotide homology (86.57% predicted amino acid homology) with OtGHV4 (GenBank # MN545486). From the one SASL URO sample examined further, amplification of partial *bcl2*-like gene resulted in a 223 bp sequence after primers were edited out that had 93.27% nucleotide homology (85.13% predicted amino acid homology) with OtGHV1 (GenBank # MN334559) and 93.49% nucleotide homology (74.67% predicted amino acid homology) with OtGHV4 (GenBank # MN545486).

The SAFS samples were all considered to be OtGHV1. The virus from the SASL was considered a new virus, hereafter proposed to as Otariid gammaherpesvirus 8 (OtGHV8), but formal nomenclature from ICTV is required. Sequences were submitted to GenBank under accession numbers OR963349-57.

#### Quantitative PCR

*South American fur seals*. Descriptive results are presented in Table 2. The animal with the highest copy number in the URO swab was positive in CON and ORO swab. One other positive CON swab was positive in a URO sample, while the other 2 CON positive samples were negative in both URO and ORO samples. In the multivariable models tested, the model that explained the greatest amount of variation in OtGHV-1 prevalence in URO swabs included sex alone, but was not significant (p = 0.743). There was no significant difference in median copy number of positive swabs by sample type (p = 0.180).

*South American sea lions*. All results reflect the qPCR initially targeting the DNA polymerase gene of OtGHV1, but as described above, a different virus may be present. In the results presented below, we feel this assay is detecting both OtGHV1 and the newly proposed OtGHV8, and thus results herein for SASL are described as OtGHV1/8. Results are summarized in Table 2.

The positive samples were detected in five pups, 27 juveniles and 14 males and 18 females. In the multivariable models tested, the model that explained the greatest amount of variation in OtGHV1/8 prevalence in URO swabs included only age (p<0.0001). The odds of OtGHV1/8 detection in a subadult/adult was 10 times greater than for a pup/yearling (OR: 10.20; 95% CI: 3.31–31.25, P < 0.001). The median copy number of positive samples was significantly higher in subadults/adults than pups/yearlings (p = 0.013)

### Agreement analysis

Ninety animals had multiple sample types available (67 from all three sites, 20 from URO and ORO, and 3 from CON and ORO). There was no agreement between URO and CON swabs (Cohen’s kappa = 0.033), and between URO and ORO swabs (Cohen’s kappa = 0.027), and minimal agreement between CON and ORO swabs (Cohen’s kappa = 0.386).

#### Phylogenetic analysis

**Fig 1:**
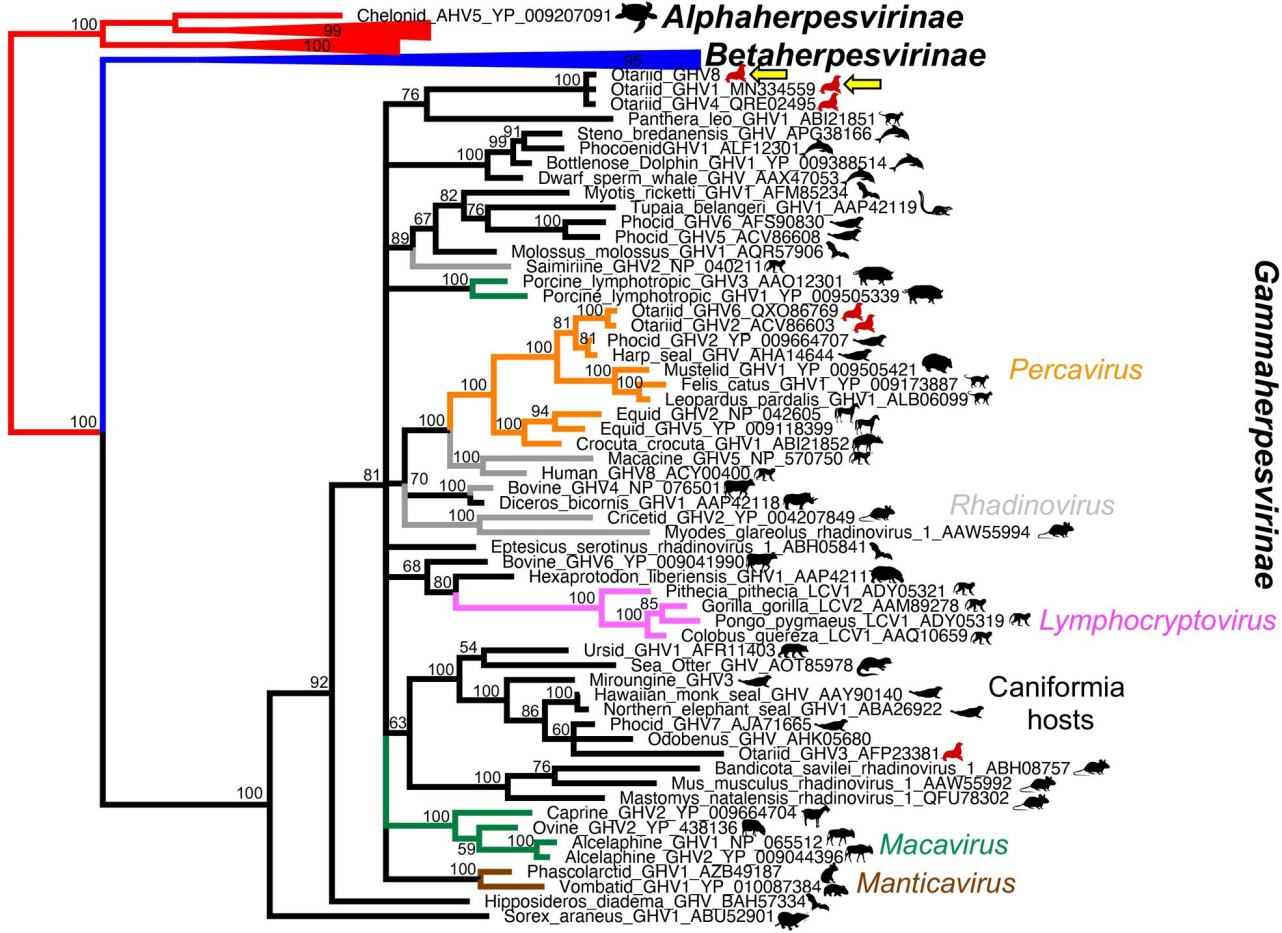
Phylogenetic tree of Otarine gammaherpesvirus DNA polymerase gene. Phylogenetic relationships of the DNA polymerase gene of Otarine gammaherpesvirus 1 (OtGHV1) and Otarine gammaherpesvirus 8 (OtGHV8) detected in South American fur seals (*Arctocephalus australis*) and South American sea lions (*Otaria byronia*), respectively, in Punta San Juan, Peru.

**Fig 2:**
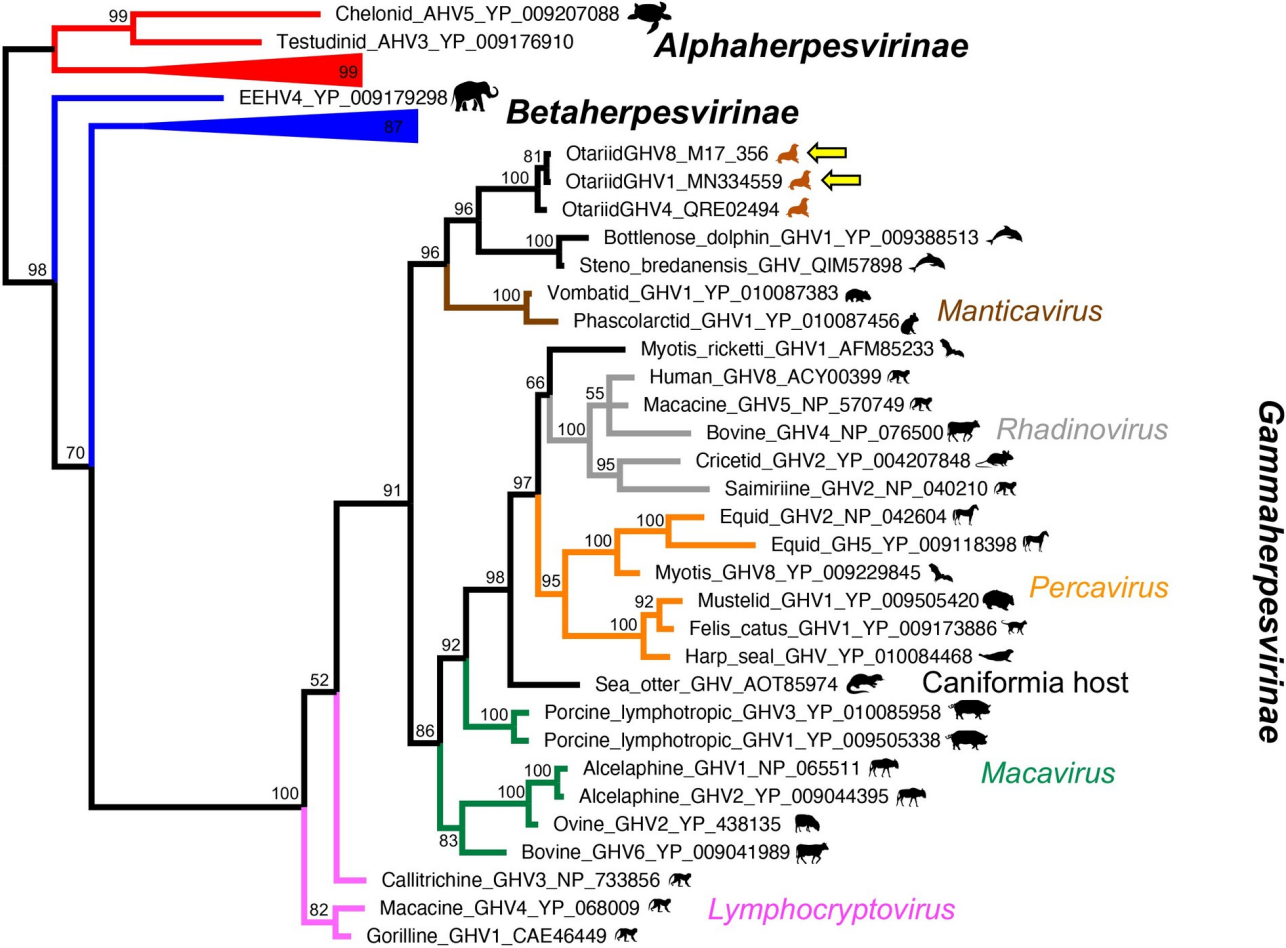
Phylogenetic tree of Otarine gammaherpesvirus glycoprotein b gene. Phylogenetic relationships of the glycoprotein b gene of Otarine gammaherpesvirus 1 (OtGHV1) and Otarine gammaherpesvirus 8 (OtGHV8) detected in South American fur seals (*Arctocephalus australis*) and South American sea lions (*Otaria byronia*), respectively, in Punta San Juan, Peru.

Bayesian model jumping found the WAG model of amino acid substitution was most probable in both the polymerase and glycoprotein B, with a posterior probability of 100% [56]. Bootstrap values from the analysis are shown on the Bayesian tree (Figs 1 and 2). OtGHV8 clustered in the subfamily Gammaherpesvirinae in both analyses with a posterior probability of 100%, but did not sit within any defined genus. Both analyses found 100% posterior probability support for a clade containing OtGHV1, OtGHV4, and OtGHV8. While the branching order within this clade could not be determined in the polymerase analysis, the glycoprotein B analysis found that OtGHV1 and OtGHV8 clustered together with a posterior probability of 81%. Although the relationships of this clade within the gammaherpesviruses were not well resolved in the analysis of the polymerase, the glycoprotein B analysis found 96% posterior probability that this clade clustered with a clade of viruses using odontocete hosts, and 96% posterior probability that the clade of OtGHV and odontocete viruses were a sister group to the genus *Manticavirus*, which use marsupial hosts (Fig 2).

## Discussion

The original objective of this study was to determine the presence and characterize the epidemiology of herpesviruses in pinnipeds in Peru. This study is the first to detect OtGHV1 DNA and identify a novel gammaherpesvirus (OtGHV8) in wild pinnipeds in the Southern Hemisphere. Prevalence of OtGHV1in adult male SAFS (42%) and adult female SAFS (36%) were similar to those observed in CSL from both the Pacific coast of California (male: 46%; female: 22%) and the Gulf of California, Mexico (female: 33%) [40, 41]. However, the discovery of OtGHV8 complicates interpretation of our qPCR results in SASL. Although the OtGHV1 qPCR was designed to be specific to OtGHV1 at the time of assay design, the close genetic relationship of the previously unknown OtGHV8 to OtGHV1 has resulted in cross-reaction in the OtGHV1 qPCR. Amplification and sequencing of three different gene targets in the SASL sample examined resulted in clean OtGHV8 chromatograms with no evidence of sequence variation at sites that differ between OtGHV1 and OtGHV8. On initial conventional PCR screening, only one of the SASL was positive, and that resulted in the discovery of OtGHV8. It is plausible, but not definitive, that all positive qPCR results in SASL were OtGHV8. Tentatively, our glycoprotein B phylogenetic analysis finds that OtGHV1 and OtGHV8 appear to be more closely related to each other than they are to OtGHV4. This matches the branching order of the hosts, regardless of whether the original endemic host for OtGHV1 are SAFS or CSL. Therefore, further investigation of gammaherpesviruses in SASL, more clearly differentiating OtGHV1 and OtGHV8, is needed.

The disparity in prevalence between the results of the conventional and qPCR results are unsurprising given the expected increased sensitivity and specificity of qPCR in relation to the nested pan-herpesvirus PCR used [39, 49]. The conventional PCR was used to screen the populations for the presence of existing and novel herpesviruses. When it was determined that OtGHV1 was present at PSJ, qPCR was pursued to increase sensitivity. All sequence data confirmed the presence of OtGHV1 in SAFS; there were no differences in the region of polymerase sequenced, as is seen intraspecies [39], and also no differences in the glycoprotein B sequence.

While it is unclear whether the qPCR results represent solely OtGHV1 or OtGHV8 or potentially a combination of OtGHV1 and OtGHV8, prevalence of virus in SASL (adult males: 47%; females: 74%) was higher in females than OtGHV1 prevalence reported in CSL, and also higher than OtGHV4 prevalence reported in female NFS (32%) [27]. The trend towards higher qPCR copy numbers in female SASL compared to males is interesting. Sexual transmission of OtGHV11 and OtGHV4 is thought to occur in CSL and NFS, respectively, which would be consistent with the higher prevalence and copy numbers of OtGHV1 and OtGHV8 found in the adult SAFS and SASL in this study, respectively, as well as the higher prevalence of positive URO swabs compared to other sample types [40]. While both male and female SAFS were sampled during their breeding seasons (October—December), only female SASL were sampled during their breeding season (January—March) [1, 2]. Male SASL were opportunistically sampled in November, which is outside their normal breeding season and may confound comparisons due to potential increased shedding during times of physiological stress. Furthermore, although the adult male SASL in this study were presumed sexually mature, they were smaller and younger males that were still likely too small to defend a territory and thus less likely to have mated with multiple females.

It is not unexpected that herpesvirus prevalence in SASL pups was significantly lower than that in adults. The prevalence of qPCR positives in SASL pups (13.5%) was higher than OtGHV1 prevalence reported in CSL pups (3.5%) in Mexico (Barragan-Vargas 2016). In one study examining the possibility of vertical transmission in CSL, a single premature pup (2.6% of sampled animals) was positive for OtHV-1, which authors concluded indicates potential for perinatal transmission in a small number of pups [40]. Most pups in this project were several weeks to a few months old based on weight and morphometric data. Future sampling of pups of varying ages may help to clarify prevalence in juvenile SASL.

The prevalence of OtGHV1 found in SAFS, along with the clinical disease found in CSL versus SAFS and the geographic phylogenetic relationships between the two species, suggest the potential for further elucidation of the definitive host of OtGHV1. The prevalence of urogenital carcinoma in CSL is one of the highest among wild mammals ever documented and this disease has been well studied for decades due to rigorous post mortem investigations [37]. Eighteen percent (66/370) of CSL necropsied from 1979 to 1994 and 12% (237/1917) of CSL necropsied from 2005–2015 were diagnosed with urogenital carcinoma [38]. Thus far, no urogenital carcinoma has been documented in any wild pinniped in Peru, although OtGHV1 has been reported in association with urogenital carcinoma in a SAFS under professional care [42]. It should be noted that available SASL and SAFS necropsy data and surveillance programs are extremely limited, especially for adult animals. In general, herpesviruses tend to cause minimal clinical disease in their host species, but are more likely to result in severe disease in aberrant hosts [57]. For example, the prevalence of Human GHV4-associated lymphoma is much higher following infection in marmoset or tamarin aberrant hosts than in human endemic hosts [58, 59]. With no evidence of urogenital carcinoma in wild SAFS in Peru, and significant evidence of urogenital carcinoma associated with OtGHV1 in CSL, particularly in California, it is therefore a consideration that SAFS may be the original endemic host for OtGHV1, with a more recent introduction into CSL.

In CSL, there are associations of host genotype with urogenital carcinoma, and MHC type is a known factor [45, 60]. Infectious diseases are the largest selective forces on vertebrates; immune-related genes show the strongest evidence of positive selection [60–63]. Introductions of disease can cause large selective sweeps on populations [64–66]. Survival is more dependent on having the right immune response to unknown future pathogens than on being stronger, faster, or smarter. The lower productivity of warmer equatorial waters has likely been a geographic barrier for otariids, and contact between species from the southern and northern hemisphere has likely not been a frequent occurrence [11]. However, recent work has demonstrated that the Peruvian fur seal is a hybridization of Galapagos fur seals and South American fur seals, thus providing a framework for possible pathogen movement between the hemispheres [16]. If a pathogen were introduced from the south, selection may have already occurred in more southern populations of CSL in the Gulf of California, where lower rates of OtGHV1 are seen, and the high rates of OtGHV1 seen in the late 1970s may represent the leading edge of spread as it travels north, followed by a decline in disease [38, 41]. Anthropogenic spread is also possible; transfer through ballast water is a potential risk that did not exist until recently [67]. Therefore, the epidemiology of OtGHV1 in CSL is consistent with a potential selective sweep after introduction from SAFS.

In NFS in the northern hemisphere, OtGHV4 appears to be endemic, and OtGHV1 has not been found in NFS despite reports of copulation between NFS and CSL, a species in which OtGHV1 is endemic [27]. A survey of Brazilian populations of SAFS and SubFS did not find the OtGHV1/4/8 clade in 23 animals [35]. The Peruvian SAFS population is genetically distinct, and may have a virome distinct from other SAFS [2, 14–16]. Further understanding of the diversity of this herpesviral clade in otariids is needed.

The SAFS OtGHV1 DNA polymerase, glycoprotein B, and *bcl2*-like gene sequences detected in this study were 100% homologous to sequences from CSL. The presence of the *bcl2*-like gene in SAFS samples shows that at least one putative oncogene is present; this gene is expressed at high levels in tumors [39]. The less conserved region of the *bcl2*-like gene was chosen with the intent of finding strain differences, while the 3’ region of the gene shows homology with diverse Gammaherpesvirinae, the 5’ region used cannot be reliably aligned with other viruses outside of the OtGHV1/4/8 clade. This region of the *bcl2*-like gene sequenced showed lower amino acid homology than nucleotide homology amongst members of the OtGHV1/4/8 clade, which is more consistent with positive selection than negative selection. However, further analysis did not detect sites of positive selection using FEL (data not shown) [68]. Genomic analysis of more viruses in the OtGHV1/4/8 clade is indicated to identify variable regions for further investigate molecular epidemiology.

In conclusion, gammaherpesviruses are present in two species of pinniped in South America, including a gammaherpesvirus associated with urogenital carcinoma in other wild pinnipeds. The complex nature of host, environment, and evolutionary pressures on herpesviruses requires further investigation in this population. Conservation actions that aim to identify emerging infectious pathogen threats strengthen the response of intervention and minimize impacts of disease in this declining population of pinnipeds [69]. Punta San Juan represents the most productive marine ecosystem in the southern hemisphere and has previously been impacted by climatic and disease events that can reduce pinniped populations, abruptly leading to genetic bottlenecks [70, 71], thus making studies such as these critical for future conservation actions. The role of host-adapted herpesvirus under these dynamic circumstances is unknown, but monitoring for changing dynamics from host-adapted (as seen with OtGHV1 at PSJ) to pathogen causing (OtGHV1 in CSLs) is certainly worth exploring. Future work may be critical to understanding the epidemiology, pathogenesis, and ecosystem health concerns in this and similar marine systems.

## Supporting information

S1 File Raw data. (XLSX)
